# Uterine myometrial mature teratoma presenting as a uterine mass: a review of literature

**DOI:** 10.1186/s12907-016-0026-8

**Published:** 2016-03-22

**Authors:** Emmanuel Kamgobe, Anthony Massinde, Dismas Matovelo, Edgar Ndaboine, Peter Rambau, Tito Chaula

**Affiliations:** Department of Obstetrics and Gynecology, Catholic University of Health and Allied sciences, P.O.BOX 1464, Mwanza, Tanzania; Department of Obstetrics and Gynecology, Bugando Medical Centre, P.O.BOX 1370, Mwanza, Tanzania; Department of Pathology, Catholic University of Health and Allied sciences, P.O.BOX 1464, Mwanza, Tanzania

**Keywords:** Uterine mass, Uterine mature teratoma

## Abstract

**Background:**

Teratomas are a germ cell tumors composed of two or more tissues which originate from ectoderm, endoderm or mesoderm. These tumors commonly arise from the ovary although other extragonadal sites can be involved, especially in children.

**Case presentation:**

We report a case of a 21-year-old female of Sukuma ethnicity from the northern region of Tanzania who presented with abdominal pain and distension, fever, and abnormal vaginal discharge for the previous three weeks. The patient was also lactating for the previous 8 months following cesarean section delivery. Pelvic ultrasound suggested pelvic abscess but after laparotomy and histological analysis of a bulky uterus removed a diagnosis of mature uterine teratoma was confirmed.

**Conclusion:**

Although it is rare, uterine teratoma should be considered in differential diagnosis to any patient with uterine mass even without typical radiological findings.

## Background

Teratomas are usually composed of two or more embryonic germ layers: ectoderm, endoderm, and mesoderm. Extragonadal teratomas are ectopic to the location in which they are found. Teratomas can be classified as mature or immature on the basis of the presence or absence of immature neuroectodermal tissues in the tumor. Mature tumors have far less tendency to develop into malignancy compared to immature tumors. Teratomas are the most common gonadal tumors. They usually arise in the gonads and often occur in infancy and childhood. Extragonadal teratomas are rare and commonly develop in midline structures [1]. Uterine teratomas, which are part of extra gonadal teratomas of midline structures, account for 1–2 % of all teratomas. Complications during their surgical removal may occur depending on their location or if they are attached to any other structures. There is no documented evidence of metastatic potential [2, 3].

Primary teratomas of the uterus have rarely been reported since Mann’s first description of this entity in 1929 [1, 2]. Here we report a case of uterine mature teratoma in a 21-year-old woman with an exceptional presentation of this tumor.

## Case presentation

A 21-year-old female of Sukuma ethnicity from the northern region of Tanzania presented at Bugando Medical Centre (BMC) outpatient clinic in Mwanza city with complaints of abdominal distension and pain, fever and abnormal vaginal discharge for the past 3 weeks. She was apparently lactating for the previous 8 months after cesarean section delivery of her first child. On physical examination, she appeared to be weak, febrile of about 38.5 °C with blood pressure of 110/70 mmHg. She was a blood group ‘A’ rhesus positive and her hemoglobin level was 6.3 g/dl.

**Fig. 1:**
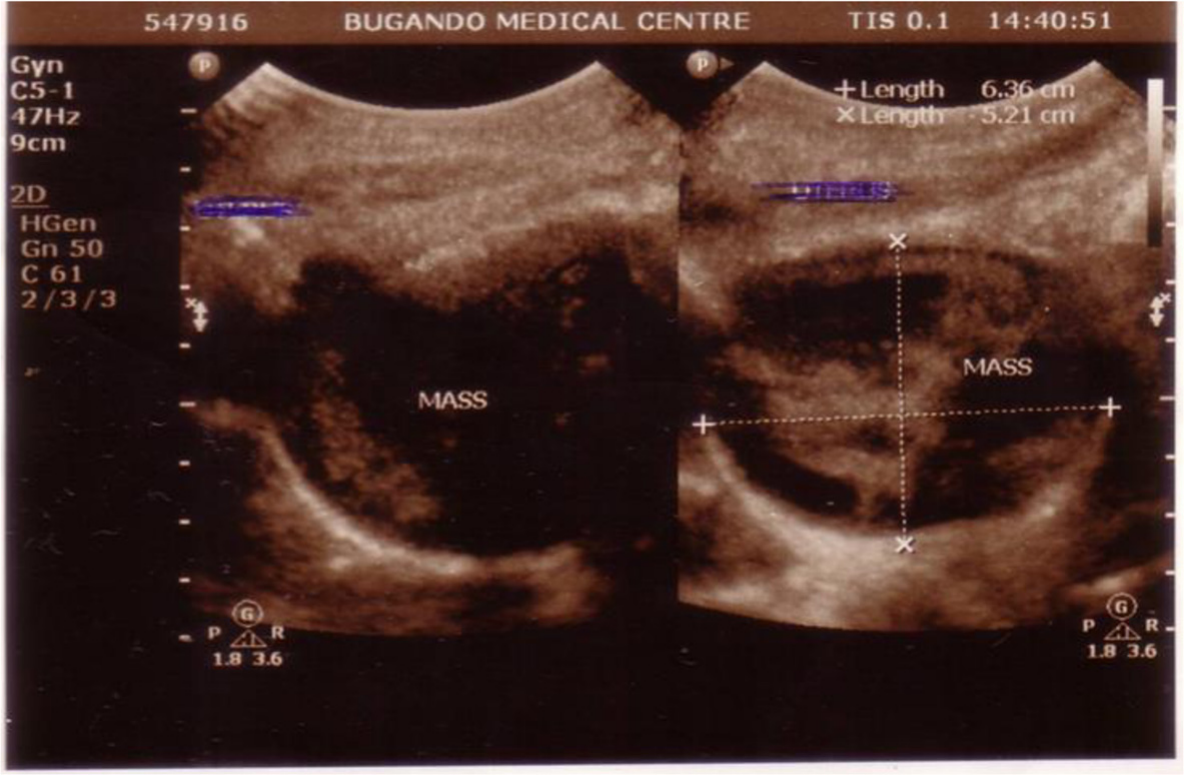
An irregular heterogeneous mass with solid echogenic areas surrounded by some fluid with no clear demarcation, measures approximately 5.3*3.4 cm, another hypoechoic mass rounded and capsulated seen posterior to the first mass predominantly cystic with some effective irregular echogenic patches measures 6.3*4.5 cm

On abdominal examination, a sub-umbilical midline scar was seen, and palpable suprapubic mass of about 16 weeks size of the uterus. The mass was soft, tender and mobile. The digital pelvic and vaginal examination elicited a closed cervix with tenderness on mobility and a non-bulging posterior fornix. There was no adnexal mass detected and gloved finger stained with pus-like discharge. Pelvic gynecological ultrasound suggested pelvic abscess (Fig. 1).

Patient was counseled for emergency laparotomy. Intraoperatively, the uterus was found to be bulky with discharging sinus on left fundal position. Both ovaries were healthy-looking and there was no fluid in the pouch of Douglas. The transverse incision was made on the uterus at the level of the discharging sinus. The yellowish mucinous tenacious materials with hairy tissues were observed. The decision to perform a total hysterectomy was reached; in which the removed uterus had hairs and sticky sebaceous matter found freely in the cavity. After surgery, the patient was transfused one unit of blood and intravenous antibiotics ceftriaxone, Gentamycin and Metronidazole were given with an addition of prophylactic Heparin. The patient had an uneventful recovery.

The sample was sent for histological examination. At the pathology department, the bisected uterus of 18 cm × 9 cm × 4 cm with no adnexa was identified. There was a cystic mass of 10 cm on the left fundal position in the myometrium containing hairs, sebaceous material, and pus.

**Fig. 2:**
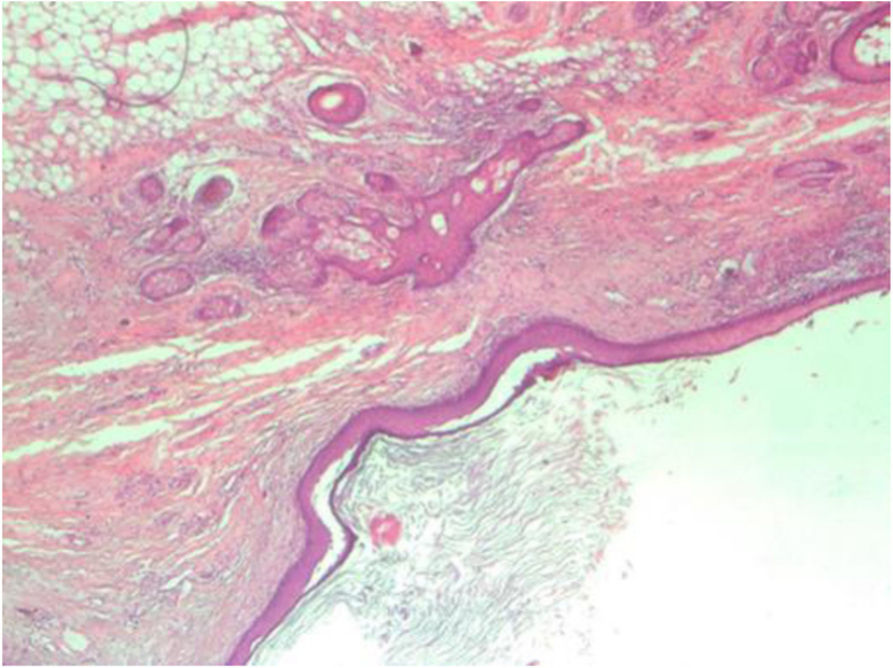
H & E histology section showing a cyst lined by squamous epithelium with keratin debris, and dermal appendages (x 10)

**Fig. 3:**
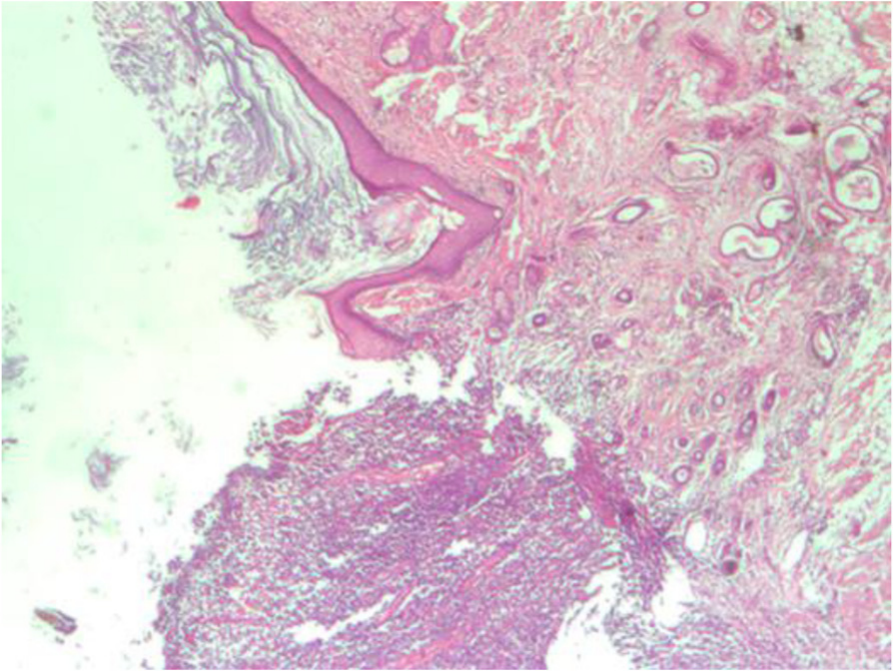
The same section showing an area of discontinuous squamous epithelium with inflammatory cells infiltrate (x10)

The tissue sample was selected and sections were stained by Hematoxylin and Eosin (H&E) and observed by a light microscopy. Histology revealed a cyst in a myometrium contained keratin debris, and it was lined by squamous epithelium with dermal skin appendages with areas of denudation with lymphocyte and neutrophil infiltrate. The myometrium and endometrium was normal. Diagnosis of infected mature teratoma (dermoid cyst) was made (Figs. 2 and 3).

The patient has not shown any sign of disease recurrence for 8 months following hospital discharge.

## Discussion

Mature cystic teratomas of uterus corpus are very rare and thought to originate from displaced germinal cells derived from pluripotential stem cells. The most commonly occurring mature cystic teratomas of the uterus are of the cervical part [2]. The presentation discussed here is unusual because there are only few reported cases of uterine teratomas to date arising from uterine body, and infected teratoma is also a rare event [2]. Extragonadal germ teratomas are very rare, accounting to 1–2 % of all teratomas [4, 5]. Since only few cases have been reported; we report the clinical presentation and pathological characteristics of patients with primary uterine mature teratoma in English literature (Table 1) [4–8].

**Table 1:** Clinical and pathological characteristics of patients with primary uterine mature teratoma in the literature

Author	Age	Symptoms	Site of Tumour	Histology	Treatment	Relapse	Treatment of Relapse
Our Case	21	Abdominal distension	Uterine corpus	Mature teratoma	Hysterectomy	None	
Lim et al., [2011] [6]	27	Cervical polyp	Uterine cervix	Mature teratoma with some lymphoid elements	Excision	None	
Newsom-Davis et al., [2009] [5]	82	Postmenopausal bleeding	Uterine corpus	Mature& Immature Teratoma	Hysterectomy & bilateral salpingooophorectomy	Yes	Taxane, Etoposide & Cisplatin + Surgery
Cappelo et al., [2009] [4]	55	Asymptomatic (Multiple uterine leiomyomas)	Uterine corpus	Mature teratoma with thyroid differentiation	Hysterectomy	None	
Wang et al., [2011] [7]	46	Abnormal uterine bleeding	Uterine corpus	Mature cystic teratoma	Hysterectomy	None	
Papadia et al., [2007] [8]	58	Endometrial polyp/Abnormal uterine bleeding	Uterine corpus	Mature cystic teratoma	Excision	None	

Our patient was in reproductive age, recently delivered and lactating; the presentation which is likely to develop teratoma due to the possibility of products of conceptus being implanted at any part along the reproductive tract during the process of fertilization according to the Blastomere Theory [8–10]. Although it does not apply to the patient discussed here, it is also possible for teratoma to develop in a newborn by abnormal migratory pathway of primordial germ cells from fetal yolk sac endodermal to the gonadal ridge during early embryogenesis as explained by Parthenogenic theory [3, 5].

Ultrasound images of the patient suggested pelvic abscess, but an explorative laparotomy and histopathological analysis of the excised bulky uterus led to a diagnosis of teratoma. In centers with, CT and MRI, those imaging techniques could have led to a correct diagnosis because of their higher accuracy compared to the ultrasound imaging [5]. Again, our hospital do not have Colour Doppler ultrasound which is superior to other imaging modalities with highest positive predictive value of 9.6 compared to 7.6 for MRI and 3.6 for CT16 [11].

Due to its rarity of occurrence, there is no standard guideline for management of mature uterine teratoma. The few case reports, however, suggest that the best management is complete tumor excision or total abdominal hysterectomy [3, 4, 6–8].

## Conclusion

Although it is rare, uterine teratoma should be considered in differential diagnosis in any patient with uterine mass even without typical radiological findings.

### Consent

"Written informed consent was obtained from the patient for publication of this Case report and any accompanying images. A copy of the written consent is available for review by the Editor of this journal".

## Abbreviations

BMC: Bugando Medical Centre
H&E: Hematoxylin & Eosin
